# 195. Reducing Time to Optimal Antimicrobial Therapy (OAT) for Bloodstream Infections (BSI) due to Gram Positive Cocci (GPC) in Chains Using Rapid Diagnostics Paired with Antimicrobial Stewardship (ASP) Efforts

**DOI:** 10.1093/ofid/ofac492.273

**Published:** 2022-12-15

**Authors:** Justin K Ma, Terrence D McSweeney, Dayna McManus, Matthew W Davis, Jacob Merwede, David Peaper, Jeffrey E Topal

**Affiliations:** Kaweah Health Medical Center, Tulare, California; Montefiore Medical Center, Massapequa, New York; Yale New Haven Hospital, New Haven, Connecticut; Yale New Haven Hospital, New Haven, Connecticut; Yale New Haven Hospital, New Haven, Connecticut; Yale University, New Haven, Connecticut; Yale New Haven HospitalYale University School of Medicine, New Haven, Connecticut

## Abstract

**Background:**

Rapid microbiology diagnostic results paired with immediate ASP intervention have been shown to decrease time to OAT and improve patient outcomes. For Gram positive (GP) BSI, studies have primarily focused on Staphylococci. However, the value of using rapid diagnostics for GPC in chains is not well studied. With the use of BioFire® FilmArray® Blood Culture Identification 2 (BCID2) to identify GPC in chains, we aimed to compare the time to OAT between pre- and post- implementation.

**Methods:**

In May 2021, our institution implemented the use of BCID2 for GPC in chains, which detects 5 types of GPC in chains to the species level and the *van A/B* gene. Positive results were communicated in real time 24/7 to ASP who provided recommendations on OAT to the treatment team.

A retrospective chart review compared the pre-implementation cohort (Jan - Sept 2019) to the post-implementation cohort (May 2021 - Jan 2022). The following patients were excluded from analysis: < 18 years of age, presence of polymicrobial infection or Streptococci with unidentified species by BCID2, discharged or deceased before rapid diagnostic result, on comfort measures or left against medical advice within 72 hours of result.

The primary study endpoint was time to OAT which was defined as time from positive Gram stain to OAT. Secondary outcomes included percentage of ASP recommendations accepted, length of stay (LOS), and 30-day mortality.

**Results:**

A total of 199 patients met inclusion criteria, 117 pre-cohort vs. 82 post-cohort. Baseline demographics were similar in both groups with the exception of the post-cohort having a numerically higher Charlson Comorbidity Index (Table 1). The most common sources of the bacteremias were intra-abdominal, skin and soft tissue, and endovascular (Table 2).

The primary endpoint, average time to OAT, was 35 hours in the pre-cohort and 18 hours in the post-cohort with a difference of 16.8 hours (95% CI 9.8 – 23.8; p < 0.0001) (Table 3). ASP recommendations were accepted 91% of the time. The median LOS was 9 days (IQR 6 - 21) in the pre-cohort vs. 13 days (IQR 7-32) in the post-cohort (p = 0.07). The 30-day mortality rate was numerically lower in the pre-cohort (6.1% vs. 10.3%; p = 0.79).

**Figure:**
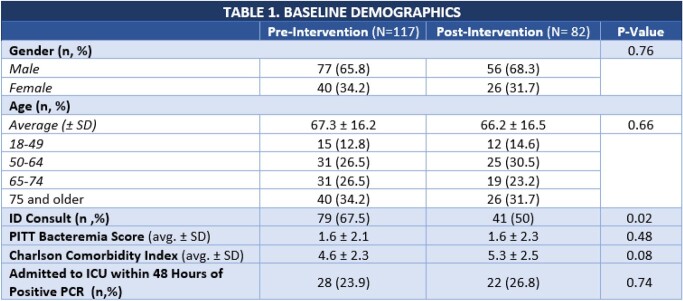

**Figure:**
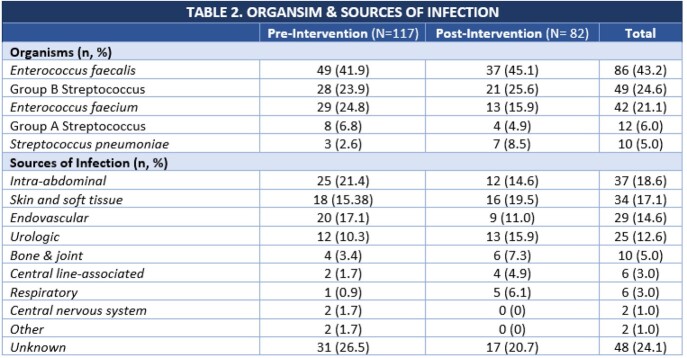

**Figure:**
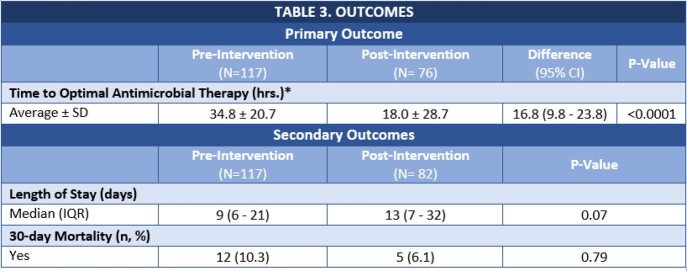

**Conclusion:**

For BSI due to GPC in chains, rapid diagnostics via BCID2 paired with 24/7 ASP led to markedly decreased time to OAT.

**Disclosures:**

**David Peaper, MD**, Tangen Biosciences: Stocks/Bonds.

